# Conceptual Design and Image Motion Compensation Rate Analysis of Two-Axis Fast Steering Mirror for Dynamic Scan and Stare Imaging System

**DOI:** 10.3390/s21196441

**Published:** 2021-09-27

**Authors:** Jianjun Sun, Yalin Ding, Hongwen Zhang, Guoqin Yuan, Yuquan Zheng

**Affiliations:** 1 Key Laboratory of Airborne Optical Imaging and Measurement, Changchun Institute of Optics, Fine Mechanics and Physics, Chinese Academy of Science, Changchun 130033, China; sunjianjun@ciomp.ac.cn (J.S.); zhanghongwen@ciomp.ac.cn (H.Z.); yuanguoqin@ciomp.ac.cn (G.Y.); 2 University of Chinese Academy of Sciences, Beijing 100049, China; 3 Changchun Institute of Optics, Fine Mechanics and Physics, Chinese Academy of Sciences, Changchun 130033, China; zhengyq@sklao.ac.cn

**Keywords:** dynamic step and stare, fast steering mirror (FSM), image motion compensation (IMC), coordinate transformation, aluminum mirror, flexural pivot

## Abstract

In order to enable the aerial photoelectric equipment to realize wide-area reconnaissance and target surveillance at the same time, a dual-band dynamic scan and stare imaging system is proposed in this paper. The imaging system performs scanning and pointing through a two-axis gimbal, compensating the image motion caused by the aircraft and gimbal angular velocity and the aircraft liner velocity using two two-axis fast steering mirrors (FSMs). The composition and working principle of the dynamic scan and stare imaging system, the detailed scheme of the two-axis FSM and the image motion compensation (IMC) algorithm are introduced. Both the structure and the mirror of the FSM adopt aluminum alloys, and the flexible support structure is designed based on four cross-axis flexural hinges. The Root-Mean-Square (RMS) error of the mirror reaches 15.8 nm and the total weight of the FSM assembly is 510 g. The IMC rate equations of the two-axis FSM are established based on the coordinate transformation method. The effectiveness of the FSM and IMC algorithm is verified by the dynamic imaging test in the laboratory and flight test.

## 1. Introduction

Aerial reconnaissance cameras generally have two working modes: wide-area reconnaissance/search and target surveillance/tracking. In the wide-area reconnaissance/search mode, the camera can obtain high-resolution images of the target area efficiently, which requires the aerial reconnaissance cameras to have the characteristics of high resolution and wide coverage. In the surveillance/tracking mode, the camera can obtain the target image and trajectory in real time, which requires the aerial reconnaissance camera to have the characteristics of a high frame frequency and positioning accuracy [1,2,3,4,5]. There are four common aerial camera imaging modes: staring imaging, linear array scanning imaging, step and stare imaging, and dynamic scan and stare imaging [6,7,8,9,10,11]. The advantages and disadvantages of these imaging methods are shown in Table 1. Obviously, the dynamic scan and stare imaging system can fully utilize the advantages of a small-array detector with a high frame frequency, improve scanning efficiency and expanding the imaging field of view (FOV). It can be used for both wide area reconnaissance and target surveillance. However, dynamic scan and stare imaging introduces image motion caused by high-frequency scanning. If the image motion cannot be compensated, the imaging resolution will significantly decrease. Therefore, it is necessary to introduce an effective IMC method.

The essence of compensating the image motion of the optical facility is to keep the relative stillness between the image of the target and the photosensitive medium during the exposure time. According to the factors that cause the image motion, the image motion can be divided into the following categories: the forward image motion caused by the flight velocity of the aircraft, the random image motion caused by the aircraft altitude motion, the scanning image motion caused by the optical facility scanning process, and the vibration image motion caused by other mechanical vibration. For the vibration image motion, shock absorbers are generally used to reduce its impact on imaging, while the other image motion must be compensated actively. Common IMC methods include moving the photosensitive medium, the electronic method, the image processing method and rotating/moving optical elements [7,11,12,13,14,15,16,17,18,19,20].

The method of moving the photosensitive medium is to keep the moving speed of the photosensitive medium matched with the speed of image motion, so as to eliminate the image motion [11]. Different from detectors with small pixels [12], such as mobile camera detectors, the charge-coupled device (CCD) or complementary metal oxide semiconductor (CMOS) image sensor used for the aerial camera usually has a larger pixel size and is relatively heavy, so a special mechanism needs to be designed to improve the compensation accuracy and control bandwidth for the dynamic scan and stare imaging system. In addition, the movement distance of the mechanism should be large enough to ensure a sufficient compensation range. This mechanism scheme is not yet fully developed.

The electronic method of IMC is to match the linear velocity of charge transfer with the linear velocity of image movement during exposure through the charge transfer technology. At present, this is mainly used in linear array time delayed and integration (TDI) CCD cameras [13]. Area-array CCD or CMOS image sensors with the time delay integration function can also be applied to an aerial camera. However, due to the long time taken for the charge readout and the low frame frequency, it cannot achieve real-time surveillance of a target and does not meet the requirements for the dynamic scan and stare imaging system.

The image processing method of motion compensation is based on the image degradation model deblurring the images, which has the advantage of low cost and strong flexibility [15,16]. However, the dynamic scan and stare imaging system has a large amount of data, so the processing time increases and the real-time performance is poor. The image processing method does not meet the requirements of dynamic scan and stare imaging system.

The method of rotating/moving optical elements of IMC is to keep image stationary relative to the imaging medium during the exposure by changing the direction of the incident light [17,18,19,20]. Traditional applications include compensating the image motion caused by the flight velocity and attitude motion of the aircraft through a stable gimbal or by adding a scanning mirror in front of the optical system to compensate the image motion caused by the flight velocity and pitch motion of the aircraft. However, due to the large rotational inertia of the compensation mechanism (the stable gimbal or scanning mirror), the compensation accuracy and bandwidth are relatively low. In order to reduce the rotational inertia of the compensation mechanism, a light and small mirror, which is usually called FSM, can be set inside the optical path to control the light deflection, so as to realize IMC with a high precision and bandwidth. Therefore, the IMC of the dynamic scan and stare imaging system based on FSM is a preferred option.

At present, scholars have carried out the design and development of FSM with different structure forms [21,22,23,24,25,26,27,28,29,30], and applied it (single-axis or two-axis) to light direction control [31,32,33,34]. In particular, Chang et al. presented the concept of a four-degrees-of-freedom (DOF) FSM based on double Porro prisms to compensate 4-DOF laser errors [35] and designed a 4-DOF actuator to implement the idea [36]. However, these studies focus more on the line of sight (LOS) stabilization algorithm and control method [37,38,39]. Although Xiu et al. focus on the IMC algorithm, their work is based on two single-axis FSMs [34]. Moreover, there are differences in the compensation algorithms for different structural forms of FSM.

This paper focuses on the structure implementation of the FSM and a high-precision IMC algorithm. In the second part, the composition and working principle of the dynamic scan and stare imaging system are introduced. The design of a two-axis FSM and its compensation rate equations and analysis process are introduced in Section 3 and Section 4. The results of a dynamic imaging test in the laboratory and flight test are analyzed in Section 5 and Section 6 summarizes all the work.

## 2. Dynamic Scan and Stare Imaging System

**Figure 1:**
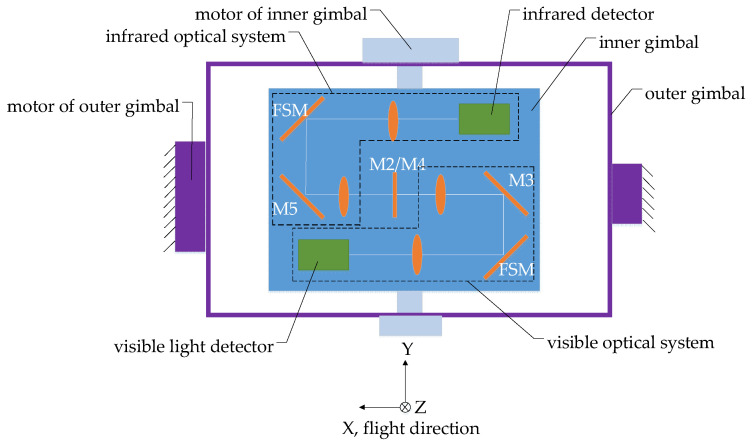
Composition of the dynamic scan and stare imaging system.

**Figure 2:**
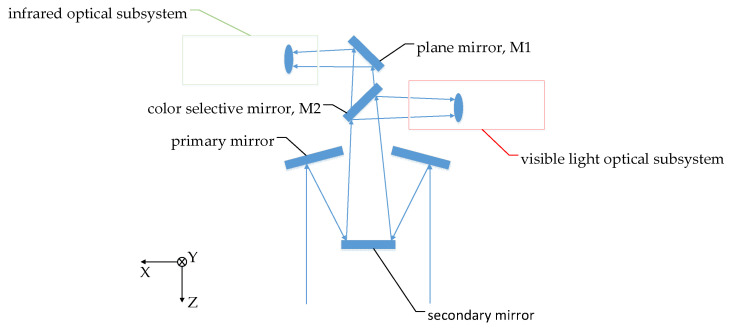
Schematic diagram of optical system.

**Figure 3:**
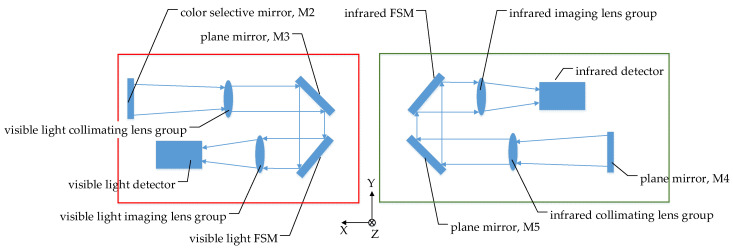
Visible light (**left**)/infrared (**right**) subsystem.

The dynamic scan and stare imaging system studied in this paper is composed of a two-axis gimbal, optical subsystem and electronic control subsystem, as shown in Figure 1. The gimbal is used to achieve the LOS pointing and expand the imaging FOV through scan motion. The optical subsystem is installed on the inner gimbal and adopts a visible light/medium infrared, dual-band, shared-aperture optical design, including a primary mirror, secondary mirror, color-selective mirror, visible light/infrared collimating lens groups, visible light/infrared imaging lens group, and mirrors used for turning the light path and 2 FSMs, as shown in Figure 2 and Figure 3. The electronic control subsystem is used to realize the motion control and imaging control, etc. The FSMs are used to compensate the image motion caused by the flight speed, attitude motion and the gimbal scanning. Although compensation can be achieved by using a two-axis FSM or two single-axis FSMs in each imaging subsystem, two two-axis FSMs are adopted, which are set in the visible light and infrared subsystem, respectively, due to the limited space.

**Figure 4:**
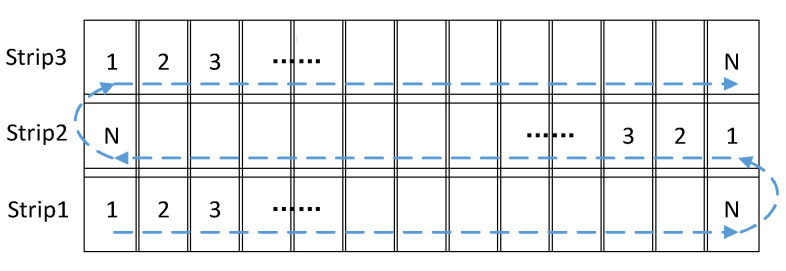
Working principle of the imaging system.

The working principle of the imaging system is shown in Figure 4. The system forms one strip through scanning imaging and forms the second strip by scanning in the opposite direction after the prior strip is finished. The overlapping ratio of the two adjacent strips is not below 10%. Through continuous scanning imaging, a large and high-resolution mosaic image of the target area can be formed. When the system performs wide-area reconnaissance/search missions, the system performs maximum-angle scanning imaging to cover the largest area in the shortest time. When the system performs surveillance/tracking tasks in a small and specific area, it only scans in a small range to obtain more exposure time. 

**Figure 5:**
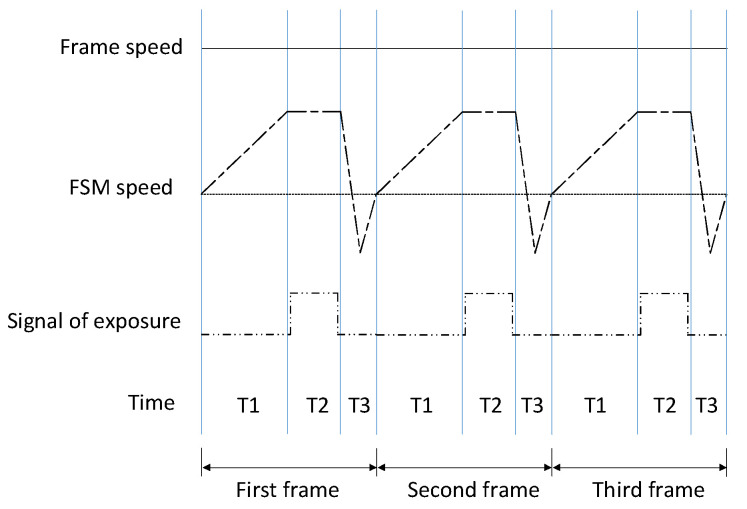
Timing/velocity relationships between the gimbal and the FSM; Timing diagram of the exposure trigger signal during imaging.

The timing/velocity relationships between the gimbal and the FSM, and the timing diagram of the exposure trigger signal during imaging is shown in Figure 5. The gimbal rotates continuously at a constant angular velocity during the work. Within one frame of imaging time, the FSM takes T1 time to accelerate to the compensation angular velocity, calculated by the command unit, to maintain stabilization. Then, the command system controls the detector exposure in T2 period. After one exposure, the FSM will return to the initial position within T3 time to prepare for the next exposure. Although FSM accelerates in both TI and T3 period, the T1 time is generally longer as the FSM has to reach a stable speed before the exposure. The acceleration of the FSM is greater during the T3 period. As the exposure time of infrared detector is longer (up to 20 ms) than the visible light detector, the T1 and T3 time of the infrared subsystem is shorter than the visible light subsystem when the imaging frame frequency is constant. So the requirement for the acceleration performance of the FSM in the infrared subsystem is higher.

## 3. FSM Design and Implementation

### 3.1. Working Principle and Design Requirements

**Figure 6:**
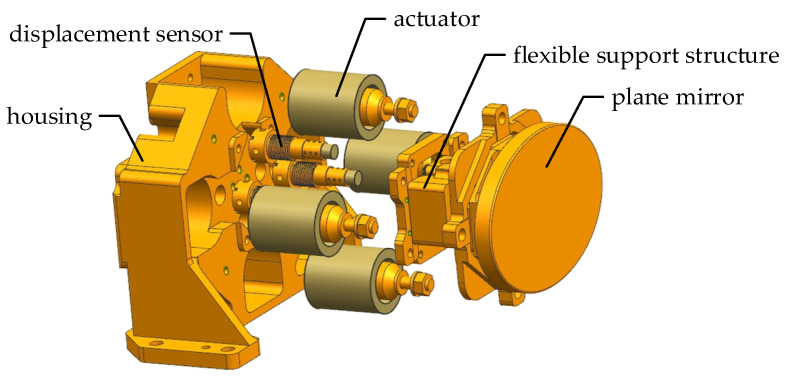
Structure composition of two-axis FSM.

The IMC frequency is high as the dynamic scan and stare imaging system has a high imaging frame frequency. Therefore, it requires the compensation mechanism to have a high response speed, positioning accuracy and closed-loop bandwidth. Figure 6 shows the structure composition of the two-axis FSM. The FSM is mainly composed of a plane mirror, flexible support structure, actuators, displacement sensors, a housing and the controller. After being pushed and pulled by the actuators, the plane mirror produces a two-dimensional small-angle rotation, relying on the elastic deformation of the flexural hinges in the flexible support structure. The displacement sensors measure the displacement of the plane mirror in real time to realize the indirect measurement of the rotation angle. Then, the closed-loop control of the FSM can be completed.

According to the performance requirements of the dynamic scan and imaging system studied in this paper, the main performance requirements of the two-axis FSM are shown in Table 2.

### 3.2. The Plane Mirror Design and Fabrication

The plane mirror is the payload of the FSM. Firstly, a sufficiently high surface profile accuracy is required for the whole working process to ensure the imaging quality. For the infrared FSM, the RMS error is required to be greater than 60 nm. For the visible light FSM, the RMS error is required to be greater than 18 nm. In order to reduce the performance requirements of the actuators, the plane mirror is required to have as small a rotational inertia as possible.

**Figure 7:**
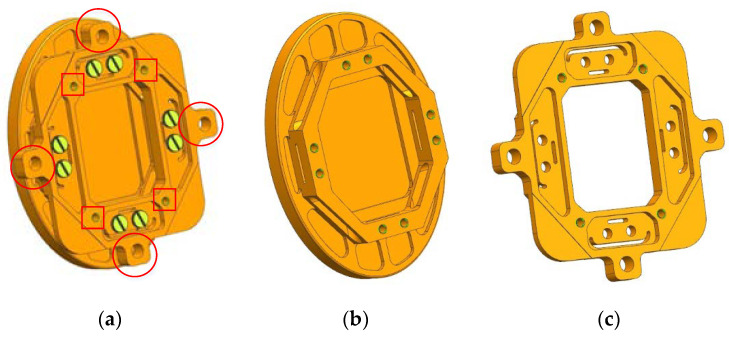
Outline of mirror: (**a**) Assembly; (**b**) Mirror body; (**c**) Backplane.

Since the mirror needs to be connected with the actuators and the flexible support structure, corresponding mechanical interfaces are reserved on the mirror. If the mirror is made of glass or SiC, it needs to be embedded in a mirror holder and the connection interface is designed on the mirror holder. This will lead to a complex structure and increase the weight and size of the FSM assembly. In order to avoid the mirror holder, RSA6061 aluminum alloys with excellent machinability are used to manufacture the mirror [40,41]. Threaded holes are machined directly onto the mirror to connect the actuators and flexible support structure. Due to the large linear expansion coefficient of the aluminum alloy, which is much different from the modified layer on the mirror surface, the bimetallic effect is easy to occur when the temperature changes. Additionally, it results in the reduction in surface profile accuracy. Therefore, the surface of the mirror is not plated with the modified layer. The mirror is polished directly on the aluminum alloy surface until the RMS error is better than 18 nm. The outline of the mirror is shown in Figure 7. In order to simplify the facilitate processing and optimize the force transmission path, the mirror was divided into two parts, the mirror body and the backplane. The two parts were connected together with eight screws before mirror polishing was carried out. Both the mirror body and the backplane were designed with lightweight grooves and stress relief holes. The thinnest position of the mirror body was only 3 mm and the total thickness was 16 mm after the mirror body was connected with the backplane. The total weight of the mirror was 82 g. The flexible support structure was connected with the mirror through the threaded hole (marked with red rectangulars in Figure 7a), and the field assemblies of voice coil motors were connected with the mirror through four cylindrical holes (marked with red circles in Figure 7a). The structure stress generated by the connection process and the self-weight of the motors was not transmitted to the mirror due to the stress relief holes of the backplane and the mirror body.

**Figure 8:**
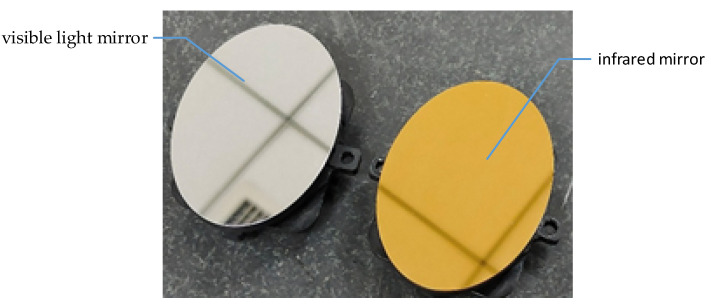
Physical objects of the visible light mirror and infrared mirror.

**Figure 9:**
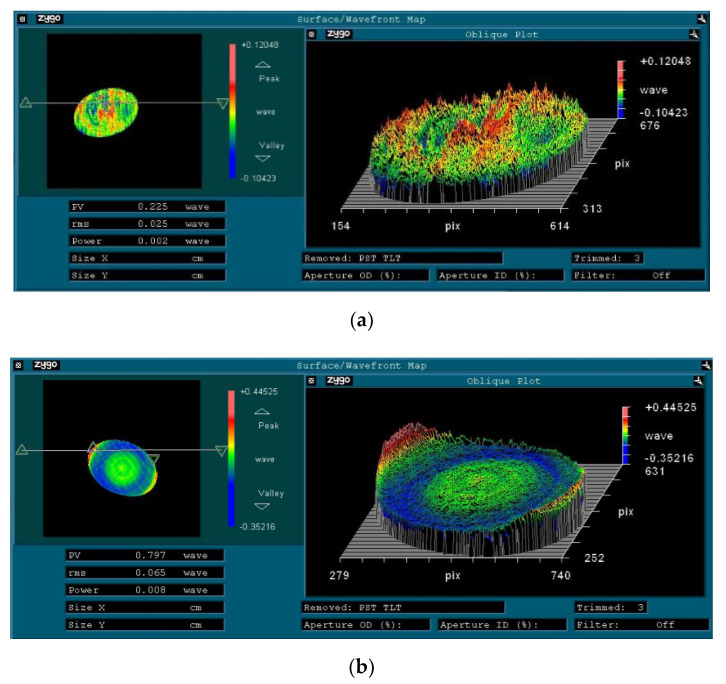
Surface profile test results; (**a**) Visible light mirror; (**b**) Infrared mirror.

The physical objects of the visible light mirror and infrared mirror are shown in Figure 8. The infrared mirror surface is plated with gold reflection film, and the visible light mirror is plated with aluminum reflection film. The results of the surface profile accuracy tested by the Zygo interferometer are shown in Figure 9 (the assembly of the FSM was completed before the test). The RMS of the visible light mirror is 15.8 nm and that of the infrared mirror is 41.1 nm. The surface profile accuracy meets the design requirements.

The results of the surface roughness test of the visible light mirror (before plating the reflection film) are shown in Table 3. A total of 16 subregions were measured. They showed that the surface roughness of the visible light mirror was greater than 3 nm.

### 3.3. Flexible Support Structure Design

The elastic deformation of the flexural hinges provides the rotational DOF for the FSM. Compared with the traditional hinge, the flexural hinge can avoid friction and backlash. Therefore, it is widely used in precision mechanics. To ensure the response speed and rotational accuracy of the FSM, the flexible hinge should have the following characteristics:

- Low rotation stiffness in the working direction;
- High rotational stiffness in the non-working direction;
- Small rotation center drift;
- Long fatigue life.

In order to avoid resonance, the first-order and second-order resonant frequencies of the two-axis FSM usually need to be much higher than the closed-loop control bandwidth of the system. The first-order and second-order vibration modes of the two-axis FSM are obviously rotating around the two working axes. If the first-order and second-order resonant frequencies are increased, the flexibility in the two axe is lost, which is contradictory to the flexible support structure design. As the resonance of the FSM in the two working directions can be eliminated by introducing the speed feedback loop into the controller, and considering reducing the control difficulty, the resonant frequencies in the two working directions (the first-order and second-order resonant frequencies) are designed to be much lower than the closed-loop bandwidth. The resonance in the non-working direction cannot be eliminated by the controller, so the third-order and the above-resonance frequencies are designed to be much higher than the closed-loop bandwidth. Generally, f1≤ωb4,f2≤ωb4 and $f_{3}\geq \sqrt{2}\omega_{b}$ are required, where $f_{1}$ , $f_{2}$ and $f_{3}$ are the first three order frequencies of the FSM and $\omega_{b}$ is the closed-loop bandwidth of the FSM.

**Figure 10:**
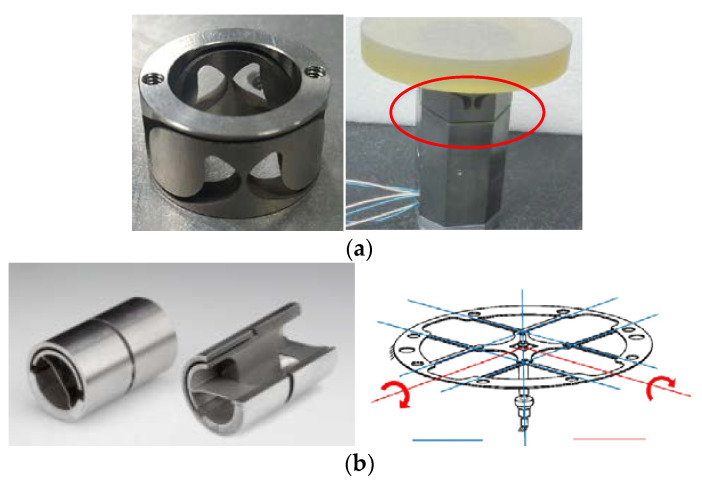
Flexural hinge; (**a**) Notch type; (**b**) Tape-spring type.

**Figure 11:**
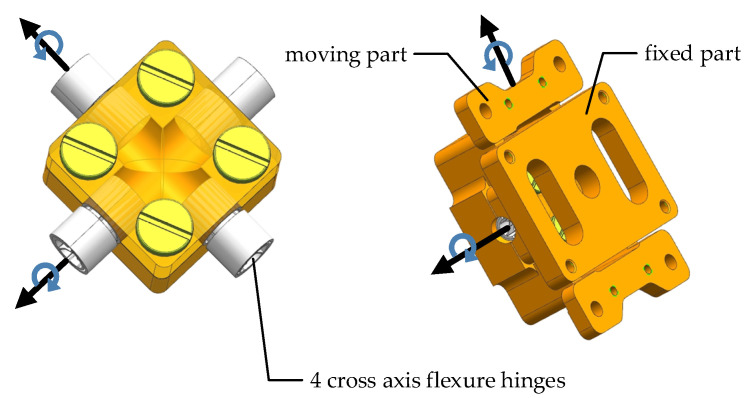
Flexible supporting structure.

The common types of flexural hinges in FSM include the notch type and tape-spring type, as shown in Figure 10. The notch type flexural hinges generally have a small angle range (generally ±0.5°~±0.6°). However, tape-spring-type flexural hinges can achieve a much larger angle range [42,43,44]. For example, the cross axis flexural hinges of the Riverhawk company can achieve a ±30° angle range [45]. The angle range of the two-axis FSM studied in this paper is ±1.1°, so the notch flexural hinge cannot meet the application requirements. Therefore, the support scheme based on cross axis flexural hinges was adopted. The flexible supporting structure is shown in Figure 11. Two flexible hinges are arranged in each working direction of the flexible support structure. The moving part connected with the fast mirror can rotate around two axes relative to the fixed part connected with the housing. 

**Figure 12:**
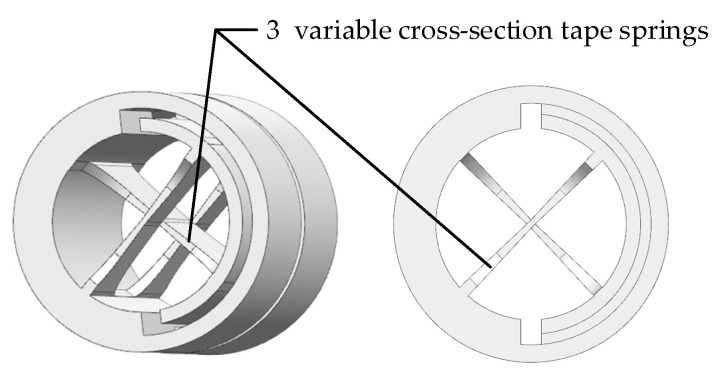
Cross axis flexural hinge with 3 variable cross-section tape springs.

The cross axis flexural hinges are specially developed for this FSM, as shown in Figure 12, with a diameter of 5 mm, an angle range of ±3.5 ° and a torsional stiffness of 0.0008 Nm/° . Different from the cross axis flexural hinge shown in Figure 10, three variable cross-section tape springs are used. The thickness of the thinnest position of the tape spring is 0.11 mm, which is half that of the thickest position.

**Figure 13:**
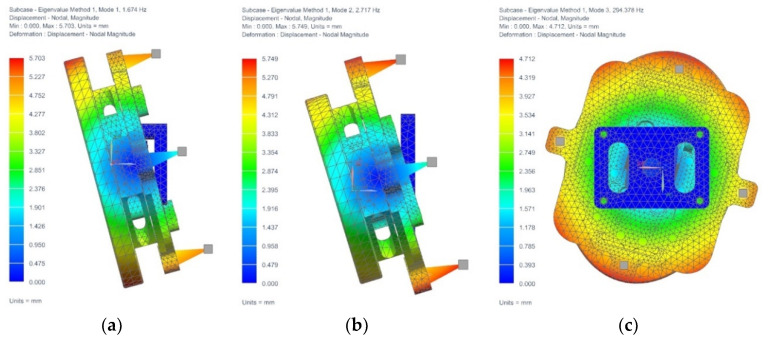
Modal analysis results; (**a**) First-order mode; (**b**) Second-order mode; (**c**) Third-order mode.

The finite element method is used to analyze the modes of the FSM and the results are shown in Figure 13. The first-order and second-order vibration modes rotate around the two working axes, and the corresponding frequencies, $f_{1}$ and $f_{2}$ , are 1.7 Hz and 2.8 Hz, respectively. The third-order vibration mode is rotation around the normal direction of the mirror and the frequency $f_{3}$ is 294.4 Hz. The first three order frequencies of the FSM meet the requirements of f1≤ωb4,f2≤ωb4 and $f_{3}\geq \sqrt{2}\omega_{b}$ . The results demonstrate that the FSM has a low rotation stiffness in the working direction and a high rotational stiffness in the non-working direction. So, the characteristics of the control bandwidth of the FSM can be realized.

### 3.4. Selection of Motor and Displacement Sensor

**Figure 14:**
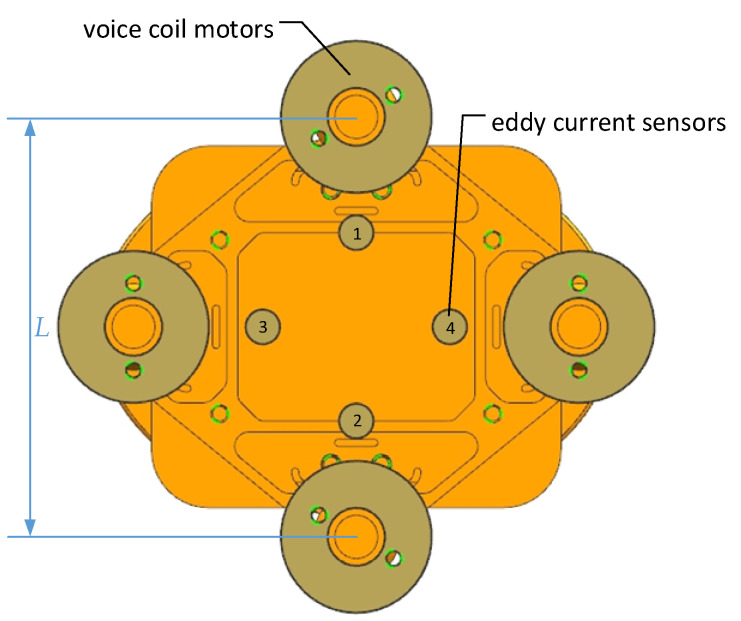
Voice coil motors and eddy current sensors arrangement scheme.

Among the actuators commonly used in FSM, voice coil motors not only have a low driving voltage, large stroke, and a fast response speed, but also have strong environmental adaptability. Voice coil motors are especially suitable for applications involving impact and vibration. According to the working environment of the aircraft and the angle range of ±1.1°, the linear voice coil motors are selected as the actuators of the FSM. In order to meet the requirements of driving torque and increase the driving stability, four voice coil motors are symmetrically arranged on the two axes, as shown in Figure 14. The driving force of the motors reaches the maximum in the start and stop process of FSM. The calculation equation of the maximum driving force is:

(1) $$F=(J\ddot{\theta }+2K\theta )/L$$

F represents the maximum driving force of the motors; K represents the torsional stiffness of the cross axis flexural hinges in working direction; θ represents the maximum rotation angle of the FSM; $\ddot{\theta }$ represents the maximum angular acceleration of the FSM; J represents the rotational inertia of the moving assemblies of the FSM; and L represents the minimum distance between the two voice coil motors in the working direction.

According to the actual design results, the values of K , θ , $\ddot{\theta }$ , J and L are 0.0008 Nm/° , 1.1°, 1500 ${\mathrm{rad}/s}^{2}$ , 0.0001 ${\mathrm{kgm}}^{2}$ , and 62 mm, respectively. Thus, it is calculated that F equals 2.49 N . Then, the motors were selected. The performances of the voice coil motors are shown in Table 4.

The non-contact eddy current sensors are selected as the displacement sensors. The eddy current sensors are also symmetrically arranged on the two rotating axes, as shown in Figure 14. This arrangement confirms that the eddy current sensors are in the best working position in the whole angle range. Additionally, it is beneficial to the measurement decoupling in the two working directions.

### 3.5. The FSM Assembly

**Figure 15:**
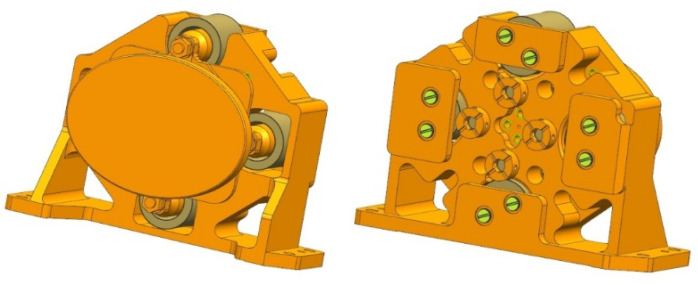
Outline of FSM.

**Figure 16:**
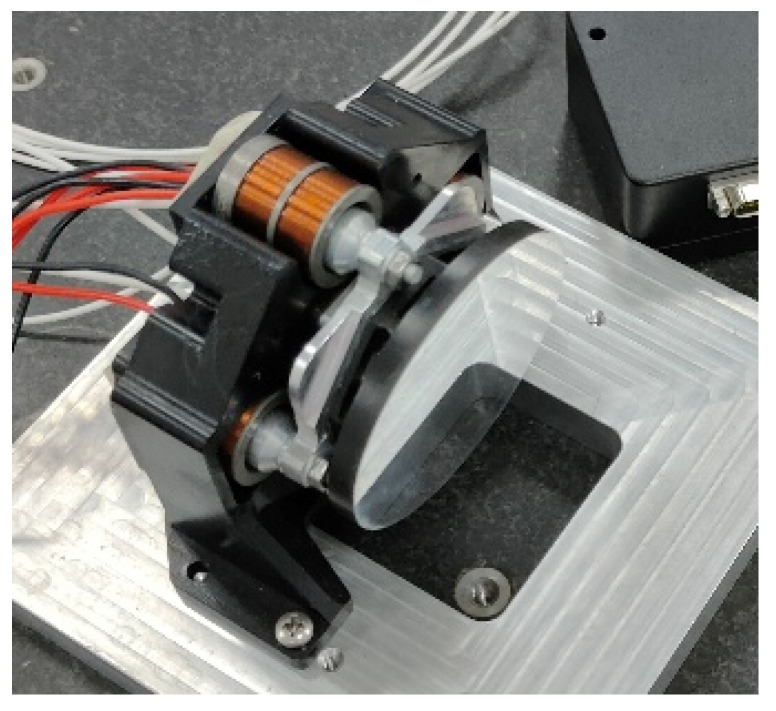
Physical object of FSM.

The outline of the FSM assembly is shown in Figure 15. All the structures of the FSM are made of aluminum alloys which have the same linear expansion coefficients as the mirror material, in order to minimize the possible thermal stress during the temperature change. The total weight of the FSM is 510 g. The physical object of the assembled FSM is shown in Figure 16.

## 4. IMC Rate Analysis

The compensation rate of the two-axis FSM is analyzed based on the coordinate transformation method. Firstly, the definition of the coordinate system used in the analysis process is clarified. All the coordinate systems conform to the right-hand rule. In addition, the compensation rate calculation methods of visible light FSM and infrared FSM are identical. Therefore, the compensation algorithms involved in the following are entirely taken from the visible light FSM, as an example.

### 4.1. Coordinate System

Geographic/Navigation coordinate system: the origin of this system is located in the natural center of the aircraft. The X axis points north along the reference ellipsoid in a meridian circle direction. The Y axis points east along the reference ellipsoid prime vertical circle direction. Additionally, the Z axis points into the ellipsoid along the normal direction of the reference ellipsoid. It is also called the north-east-down (NED) coordinate system. Navigation parameters are solved in this coordinate system. This coordinate system is represented by the symbol N and its three axes are marked as $X_{N}$ , $Y_{N}$ and $Z_{N}$ .

Aircraft coordinate system: The origin of this system is located in the natural center of the aircraft. The X axis is located in the symmetrical plane of the aircraft, parallel to the axis of the fuselage pointing forward. The Z axis is located in the symmetry plane perpendicular to the X axis pointing downward. Additionally, the Y axis is perpendicular to the symmetry plane pointing to the right. The orientation of the aircraft coordinate system relative to the geographical coordinate system is the aircraft attitude, which is expressed by three Euler angles as the heading angle ψ , pitch angle θ and roll angle ϕ . This coordinate system is represented by the symbol A and its three axes are marked as $X_{A}$ , $Y_{A}$ and $Z_{A}$ .

Gimbal coordinate system: The coordinate system is located on the inner gimbal. The origin is located at the intersection of the two axes of the inner and outer gimbal. When the rotation angle around the two-axis of the gimbal is 0, the three axes of the gimbal coordinate system and the aircraft coordinate system are parallel and share the same direction. It can be considered that the natural center of the aircraft coincides with the intersection of the two axes of the gimbal. The rotation of the gimbal around the X axis is the rolling motion of the imaging system; the rotation angle is marked as β . The rotation of the gimbal around the Y axis is the pitching motion of the imaging system; the rotation angle is marked as α . The optical subsystem is installed on the inner frame and the Z axis of the gimbal coordinate system is parallel to the primary optical axis. Therefore, the Z axis represents the direction of the LOS. This coordinate system is represented by the symbol G and its three axes are marked as $X_{G}$ , $Y_{G}$ and $Z_{G}$ .

FSM coordinate system: This coordinate system is located on the plane mirror of the FSM. The origin is at the center of the mirror. The visible light and infrared FSMs are installed on the inner gimbal. In the zero position of the FSM, the gimbal coordinate system rotates around the $Z_{G}$ axis by −45° to obtain the visible light FSM coordinate system, and rotates around the $Z_{G}$ axis by −135° to obtain the infrared FSM coordinate system. This visible light and infrared coordinate systems are represented by the symbol ME and MI .

Visible light detector coordinate system: This coordinate system is located on the inner gimbal. The origin is at the intersection of the optical axis and the detector photosensitive surface. When the rotation angle around the two axes of the gimbal is 0, the three axes of the visible light detector coordinate system are parallel to the gimbal coordinate system and share the same direction. This coordinate system is represented by the symbol I .

Visible light image coordinate system: This coordinate system is located on the inner gimbal. It entirely coincides with the visible light FSM coordinate system at the 0 position. This coordinate system is represented by the symbol $I_{0}$ and its three axes are marked as $X_{I_{0}}$ , $Y_{I_{0}}$ and $Z_{I_{0}}$ .

### 4.2. Coordinate Transformation

The geographic coordinate system can be rotated at angles of the aircraft attitude to coincide with the aircraft coordinate system. The rotation order is to rotate the heading angle ψ around the $Z_{N}$ axis, the pitch angle θ around the $Y_{N}$ axis, and the roll angle ϕ around the $X_{N}$ axis. The transformation matrix is marked as $C_{N}^{A}$ . $C_{N}^{A}$ is expressed as:

(2) $$C_{N}^{A}=\left(\begin{array}{ccc} 1 & 0 & 0\\ 0 & \cos\phi & \sin\phi \\ 0 & -\sin\phi & \cos\phi \end{array}\right)\left(\begin{array}{ccc} \cos\theta & 0 & -\sin\theta \\ 0 & 1 & 0\\ \sin\theta & 0 & \cos\theta \end{array}\right)\left(\begin{array}{ccc} \cos\psi & \sin\psi & 0\\ -\sin\psi & \cos\psi & 0\\ 0 & 0 & 1 \end{array}\right)$$

If the aircraft coordinate system is rotated at an angle of β around the $X_{A}$ axis and then at an angle of α around the $Y_{A}$ axis, then the aircraft coordinate system coincides with the gimble coordinate system. The transformation matrix is marked as $C_{A}^{G}$ . $C_{A}^{G}$ is expressed as:

(3) $$C_{A}^{G}=\left(\begin{array}{ccc} \cos\alpha & 0 & -\sin\alpha \\ 0 & 1 & 0\\ \sin\alpha & 0 & \cos\alpha \end{array}\right)\left(\begin{array}{ccc} 1 & 0 & 0\\ 0 & \cos\beta & \sin\beta \\ 0 & -\sin\beta & \cos\beta \end{array}\right)$$

Both the gimbal coordinate system and visible light image coordinate system are located on the inner gimbal. The gimbal coordinate system can be rotated at an angle of −45° around its $Z_{G}$ axis to coincide with the visible light image coordinate system. The transformation matrix is marked as $C_{G}^{I_{0}}$ . $C_{G}^{I_{0}}$ is expressed as:

(4) CGI0=(cos(−45°)sin(−45°)0−sin(−45°)cos(−45°)0001)=(22−22022220001)

According to Snell’s Law, after the incident light (the incident light vector is denoted as $P_{in}$ ) is reflected by a mirror (the normal vector is denoted as N ), the outgoing light vector (denoted as $P_{out}$ ) can be expressed as:

(5) $$P_{out}=T\times P_{in}$$

(6) $$T=\left(\begin{array}{ccc} 1-2N_{X}^{2} & -2N_{X}N_{Y} & -2N_{X}N_{Z}\\ -2N_{X}N_{Y} & 1-2N_{Y}^{2} & -2N_{Y}N_{Z}\\ -2N_{X}N_{Z} & -2N_{Y}N_{Z} & 1-2N_{Z}^{2} \end{array}\right)$$

(7) $$N={\left(N_{X},N_{Y},N_{Z}\right)}^{T}$$

In the visible light image coordinate system, the incident light (the primary optical axis vector is denoted as $P_{I_{0}in}$ ) passing through the primary mirror and the secondary mirror can reach the visible light detector after being reflected by the M2 plane mirror, M3 plane mirror and the visible light FSM (normal vectors are marked as $N_{2}$ , $N_{3}$ and $N_{ME}$ , respectively). The primary optical axis vector of outgoing light reflected by the FSM is marked as $P_{I_{0}out}$ . $P_{I_{0}out}$ is expressed as:

(8) $$P_{I_{0}out}=T_{ME}\times T_{3}\times T_{2}\times P_{I_{0}in}$$

In the visible light image coordinate system, the normal vectors of the M2 and M3 plane mirror are determined. Therefore, the corresponding matrices, $T_{2}$ and $T_{3}$ , are also determined. They are:

(9) N2=(N2X,N2Y,N2Z)T=(−12,12,22)T

(10) $$N_{3}={\left(N_{3X},N_{3Y},N_{3Z}\right)}^{T}={\left(0,-1,0\right)}^{T}$$

(11) T2=(1212221212−2222−220)

(12) $$T_{3}=\left(\begin{array}{ccc} 1 & 0 & 0\\ 0 & -1 & 0\\ 0 & 0 & 1 \end{array}\right)$$

The normal vector of the FSM moves with the rotation of the FSM. The rotation angles of the visible light FSM around the vertical axis and the horizontal axis of the flexible support structure are marked as λ and δ , and the positive directions of the vertical axis and horizontal axis of the flexible support structure are defined as the directions at an acute angle to the positive directions of the $Z_{I_{0}}$ axis and $Y_{I_{0}}$ . Then, the normal vector $N_{ME}$ and the corresponding matrices $T_{ME}$ of the FSM in the visible light image coordinate system can be expressed as:

(13) $$N_{ME}=\left(\begin{array}{ccc} \cos(-\delta ) & 0 & -\sin(-\delta )\\ 0 & 1 & 0\\ \sin(-\delta ) & 0 & \cos(-\delta ) \end{array}\right)\left(\begin{array}{ccc} \cos(-\lambda ) & \sin(-\lambda ) & 0\\ -\sin(-\lambda ) & \cos(-\lambda ) & 0\\ 0 & 0 & 1 \end{array}\right)\left(\begin{array}{l} 1\\ 0\\ 0 \end{array}\right)=\left(\begin{array}{l} \cos\delta \cos\lambda \\ \sin\lambda \\ -\cos\lambda \sin\delta \end{array}\right)$$

(14) $$T_{ME}=\left(\begin{array}{ccc} 1-2\cos^{2}\delta \cos^{2}\lambda & -2\cos\delta \cos\lambda \sin\lambda & 2\cos\delta \cos^{2}\lambda \sin\delta \\ -2\cos\delta \cos\lambda \sin\lambda & 1-2\sin^{2}\lambda & 2\cos\lambda \sin\delta \sin\lambda \\ 2\cos\delta \cos^{2}\lambda \sin\delta & 2\cos\lambda \sin\delta \sin\lambda & 1-2\cos^{2}\lambda \sin^{2}\delta \end{array}\right)$$

### 4.3. IMC Rate Analysis

#### 4.3.1. Compensate Image Motion Caused by the Aircraft and Gimbal Angular Velocity

Light rotates with the aircraft attitude motion, gimbal rotation and FSM rotation. The expression of the angular velocity (marked as $\omega_{M}$ ) of the primary optical axis of the outgoing light in the visible light image coordinate system is as follows:

(15) $$\begin{array}{l} \omega_{M}={\left(\omega_{MX},\omega_{MY},\omega_{MZ}\right)}^{T}=\\ T_{ME}\times T_{3}\times T_{2}\times C_{G}^{I_{0}}\times (L_{Y}(\alpha )\times (L_{X}(\beta )\times \omega_{A}+{\left(\dot{\beta },0,0\right)}^{T})+{\left(0,\dot{\alpha },0\right)}^{T})+\\ L_{Y}(-\delta )\times \left(0,0,2\dot{\lambda }\right)T+{\left(0,2\dot{\delta },0\right)}^{T} \end{array}$$

(16) $$\omega_{A}={\left(\omega_{AX},\omega_{AY},\omega_{AZ}\right)}^{T}$$

(17) $$L_{Y}(\alpha )=\left(\begin{array}{ccc} \cos\alpha & 0 & -\sin\alpha \\ 0 & 1 & 0\\ \sin\alpha & 0 & -\cos\alpha \end{array}\right)$$

(18) $$L_{Y}(-\delta )=\left(\begin{array}{ccc} \cos\delta & 0 & \sin\delta \\ 0 & 1 & 0\\ -\sin\delta & 0 & \cos\delta \end{array}\right)$$

(19) $$L_{X}(\beta )=\left(\begin{array}{ccc} 1 & 0 & 0\\ 0 & \cos\beta & \sin\beta \\ 0 & -\sin\beta & \cos\beta \end{array}\right)$$

where $\omega_{MX}$ , $\omega_{MY}$ and $\omega_{MZ}$ are the components of $\omega_{M}$ in the $X_{I_{0}}$ , $Y_{I_{0}}$ and $Z_{I_{0}}$ directions; $\omega_{A}$ is the aircraft angular velocity in the aircraft coordinate system; and $\omega_{AX}$ , $\omega_{AY}$ and $\omega_{AZ}$ are the components of $\omega_{A}$ in the $X_{A}$ , $Y_{A}$ and $Z_{A}$ directions. 

If the imaging system has no image motion, the image motion speed is 0. That is:

(20) $$\left\{ \begin{array}{l} \omega_{MY}=0\\ \omega_{MZ}=0 \end{array}\right.$$

Thus, the IMC rates $\dot{\lambda }$ and $\dot{\delta }$ can be obtained and the expressions are:

(21) $$\begin{array}{ll} \dot{\delta }= & \frac{\sqrt{2}}{2}(\cos^{2}\lambda +\cos\delta \sin\lambda \cos\lambda -\frac{1}{2})(\dot{\beta }\cos\alpha +\omega_{AX}\cos\alpha -\omega_{AZ}\cos\beta \sin\alpha +\omega_{AY}\sin\alpha \sin\beta )\\ & +\frac{\sqrt{2}}{2}(\cos\delta \cos\lambda \sin\lambda -\cos^{2}\lambda +\frac{1}{2})(\dot{\beta }\sin\alpha +\omega_{AX}\sin\alpha +\omega_{AZ}\cos\alpha \cos\beta -\omega_{AY}\cos\alpha \sin\beta )\\ & +\sin\delta \cos\lambda \sin\lambda (\dot{\alpha }+\omega_{AY}\cos\beta +\omega_{AZ}\sin\beta ) \end{array}$$

(22) $$\begin{array}{ll} \dot{\lambda }= & -\frac{1}{2}(\cos2\lambda \tan\delta \sin\delta -\cos\delta )(\dot{\alpha }+\omega_{AY}\cos\beta +\omega_{AZ}\sin\beta )\\ & -\frac{\sqrt{2}}{2}\cos\lambda \tan\delta (\sin\lambda +\cos\delta \cos\lambda )(\dot{\beta }\sin\alpha +\omega_{AX}\sin\alpha +\omega_{AZ}\cos\alpha \cos\beta -\omega_{AY}\cos\alpha \sin\beta )\\ & +\frac{\sqrt{2}}{2}\cos\lambda \tan\delta (\sin\lambda -\cos\delta \cos\lambda )(\dot{\beta }\cos\alpha +\omega_{AX}\cos\alpha -\omega_{AZ}\sin\alpha \cos\beta +\omega_{AY}\sin\alpha \sin\beta ) \end{array}$$

#### 4.3.2. Compensate Image Motion Caused by the Aircraft Liner Velocity

Light rotates with the aircraft flight. The aircraft liner velocity in the geographic coordinate system and aircraft coordinate system is marked as $V_{N}$ and $V_{A}$ . $V_{A}$ is expressed as:

(23) $$V_{A}={\left(V_{AX},V_{AY},V_{AZ}\right)}^{T}=C_{N}^{A}\times V_{N}$$

The photographic distance is marked as L . The equation for converting the aircraft liner velocity $V_{A}$ to the angular velocity $\omega_{VA}$ is as follows:

(24) $$\omega_{VA}={\left[V_{AY}/L,-V_{AX}/L,0\right]}^{T}$$

The photographic distance L at the time of exposure can be calculated by the flight altitude of the aircraft and the direction of *LOS*. The flight altitude at the time of exposure is marked as H , the *LOS* vectors in the geographic coordinate system and the gimbal coordinate system are marked as $LOS_{N}$ and $LOS_{G}$ , respectively. The expressions are as follows:

(25) $$LOS_{G}={\left(0,0,1\right)}^{T}$$

(26) $$LOS_{N}={\left(LOS_{NX},LOS_{NY},LOS_{NZ}\right)}^{T}={\left(C_{N}^{A}\right)}^{T}\times {\left(C_{A}^{G}\right)}^{T}\times LOS_{G}$$

(27) $$L=H/LOS_{NZ}$$

In this way, the image motion caused by the aircraft liner velocity can also be compensated according to Equations (21) and (22).

#### 4.3.3. Compensate Image Motion Caused by the Aircraft and Gimbal Angular Velocity and the Aircraft Liner Velocity

The variable $\omega_{TB}$ is defined as follows:

(28) $$\omega_{TB}={\left(\omega_{TBX},\omega_{TBY},\omega_{TBZ}\right)}^{T}=\omega_{B}+\omega_{VB}$$

The compensation rate of the FSM for the image motion caused by the aircraft and gimbal angular velocity and the aircraft liner velocity can be obtained according to Equations (21) and (22). The rotation angles of the FSM are marked as $\lambda_{T}$ and $\delta_{T}$ , then the compensation rates ${\dot{\delta }}_{T}$ and ${\dot{\lambda }}_{T}$ are as follows:

(29) $$\begin{array}{ll} {\dot{\delta }}_{T}= & \frac{\sqrt{2}}{2}(\cos^{2}\lambda_{T}+\cos\delta \sin\lambda_{T}\cos\lambda_{T}-\frac{1}{2})(\dot{\beta }\cos\alpha +\omega_{TBX}\cos\alpha -\omega_{TBZ}\cos\beta \sin\alpha +\omega_{TBY}\sin\alpha \sin\beta )\\ & +\frac{\sqrt{2}}{2}(\cos\delta_{T}\cos\lambda_{T}\sin\lambda_{T}-\cos^{2}\lambda_{T}+\frac{1}{2})(\dot{\beta }\sin\alpha +\omega_{TBX}\sin\alpha +\omega_{TBZ}\cos\alpha \cos\beta -\omega_{TBY}\cos\alpha \sin\beta )\\ & +\sin\delta_{T}\cos\lambda_{T}\sin\lambda_{T}(\dot{\alpha }+\omega_{TBY}\cos\beta +\omega_{TBZ}\sin\beta ) \end{array}$$

(30) $$\begin{array}{ll} {\dot{\lambda }}_{T}= & -\frac{1}{2}(\cos2\lambda_{T}\tan\delta_{T}\sin\delta_{T}-\cos\delta_{T})(\dot{\alpha }+\omega_{TBY}\cos\beta +\omega_{TBZ}\sin\beta )\\ & -\frac{\sqrt{2}}{2}\cos\lambda_{T}\tan\delta_{T}(\sin\lambda_{T}+\cos\delta_{T}\cos\lambda )(\dot{\beta }\sin\alpha +\omega_{TBX}\sin\alpha +\omega_{TBZ}\cos\alpha \cos\beta -\omega_{TBY}\cos\alpha \sin\beta )\\ & +\frac{\sqrt{2}}{2}\cos\lambda_{T}\tan\delta_{T}(\sin\lambda_{T}-\cos\delta_{T}\cos\lambda_{T})(\dot{\beta }\cos\alpha +\omega_{TBX}\cos\alpha -\omega_{TBZ}\sin\alpha \cos\beta +\omega_{TBY}\sin\alpha \sin\beta ) \end{array}$$

According to the above equations, the compensation rate of the FSM is related to the rotation angle and angular velocity of the gimbal, the aircraft angular velocity and the rotation angle of the FSM. The rotation angle and angular velocity of the gimbal can be obtained by the gyroscopes installed on the two axes. The aircraft angular velocity and flight altitude are given by the aircraft control system. The rotation angles of the FSM can be obtained in real time by the eddy current sensors. Therefore, the compensation rates ${\dot{\delta }}_{T}$ and ${\dot{\lambda }}_{T}$ of the FSM can be calculated.

Due to the magnification of the optical system, the compensation rates need to be calculated according to the magnification of the visible light and infrared subsystems based on Equations (29) and (30), respectively.

## 5. Experiments and Results

**Figure 17:**
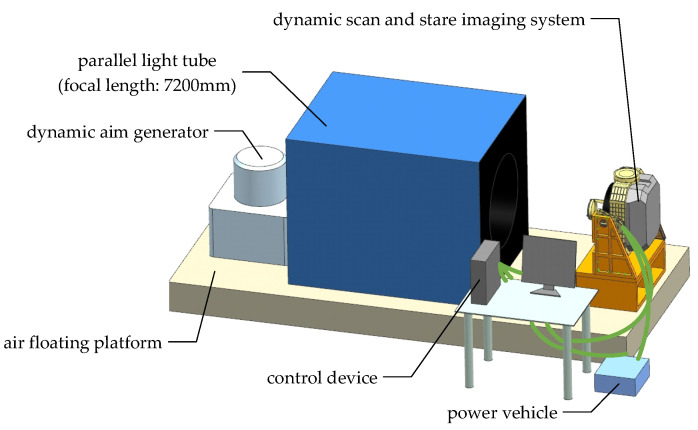
Schematic diagram of dynamic imaging test in laboratory.

**Figure 18:**
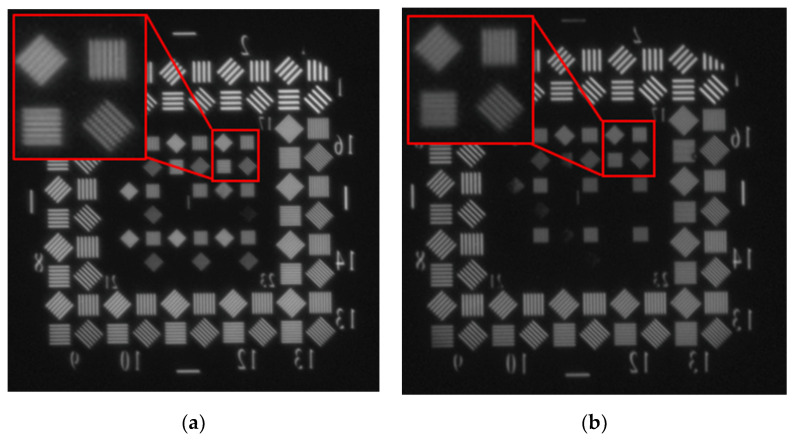

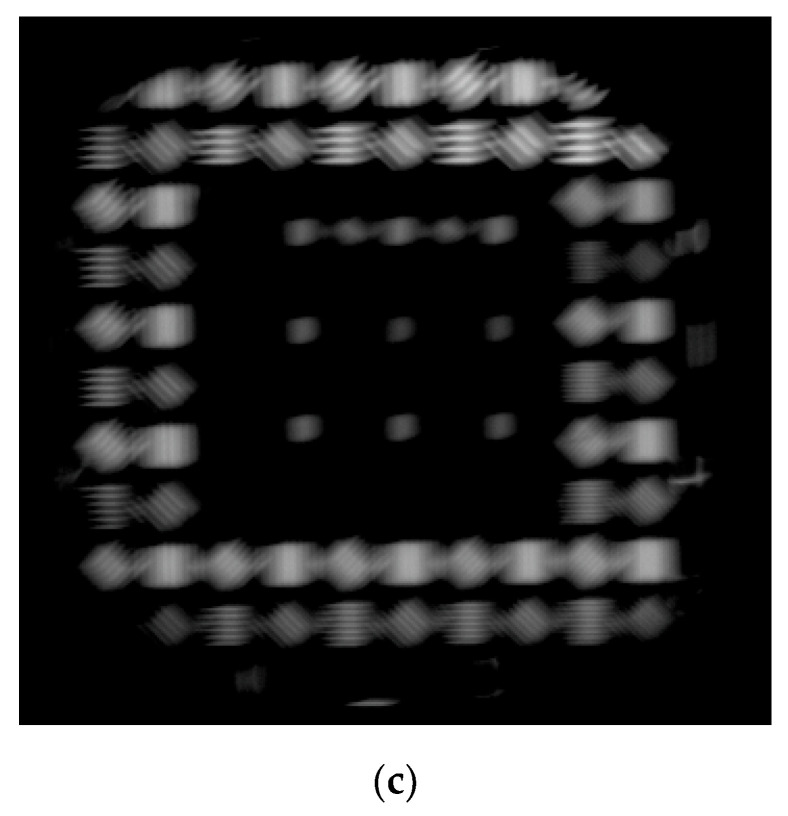
Visible light images obtained in dynamic imaging test in laboratory: (**a**) VHR = 0, $V_{G}=0{}^{\circ}/s$ ; (**b**) VHR = 0.04, $V_{G}=17.6{}^{\circ}/s$ ; (**c**) VHR = 0.04, $V_{G}=17.6{}^{\circ}/s$ and wrong IMC rates.

In order to verify the effectiveness of the FSM and IMC algorithm, the dynamic imaging test in the laboratory and flight test were carried out based on the dynamic scan and stare imaging system. The schematic diagram of the dynamic imaging test in the laboratory is shown in Figure 17. The aircraft motion at different velocity–height ratios (VHR) is accurately simulated by adjusting the rotation rate of the dynamic aim generator with a rotation radius of 150 mm. The system scanning images the dynamic target generator along the direction perpendicular to the rotation direction of the target. Additionally, the FSM compensates the image motion caused by the liner velocity of the target (aircraft) and the scanning motion of the gimbal. The scanning velocity of the gimbal is marked as $V_{G}$ . The visible light images obtained in the dynamic imaging test are shown in Figure 18. Figure 18a shows the image obtained in the test with VHR = 0 and $V_{G}=0{}^{\circ}/s$ ; Figure 18b shows the image obtained in the test with VHR = 0.04 and $V_{G}=17.6{}^{\circ}/s$ ; and Figure 18c shows the image obtained in the test with VHR = 0.04 and $V_{G}=17.6{}^{\circ}/s$ , but in which the compensation rates of the FSM were modified to the incorrect value by multiplying ${\dot{\delta }}_{T}$ and ${\dot{\lambda }}_{T}$ by 0.5 and 0.9, respectively. It can be seen that both images in Figure 18a,b can distinguish a target with a linewidth of 31.7 μm , which corresponds to the limiting resolution of the imaging system. The image becomes blurred when the IMC rates are wrong. The results prove the effectiveness of the FSM and IMC algorithm.

**Figure 19:**
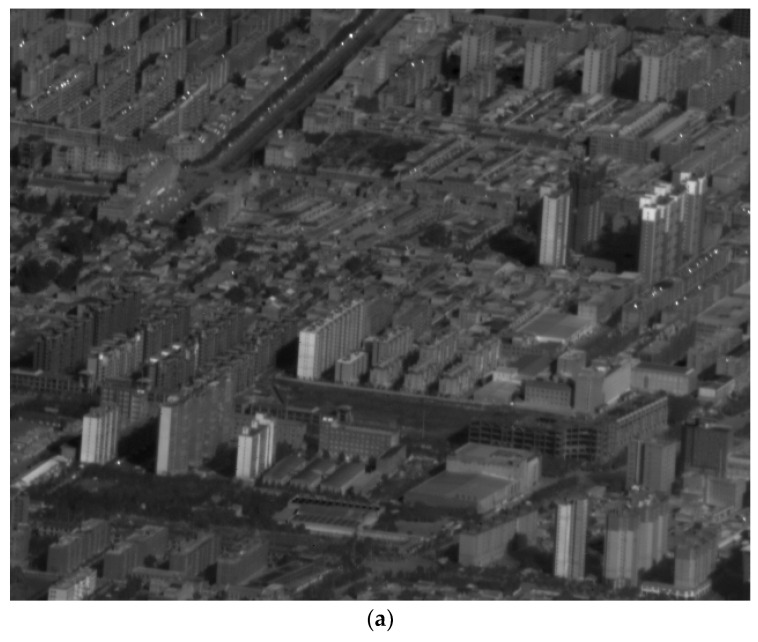

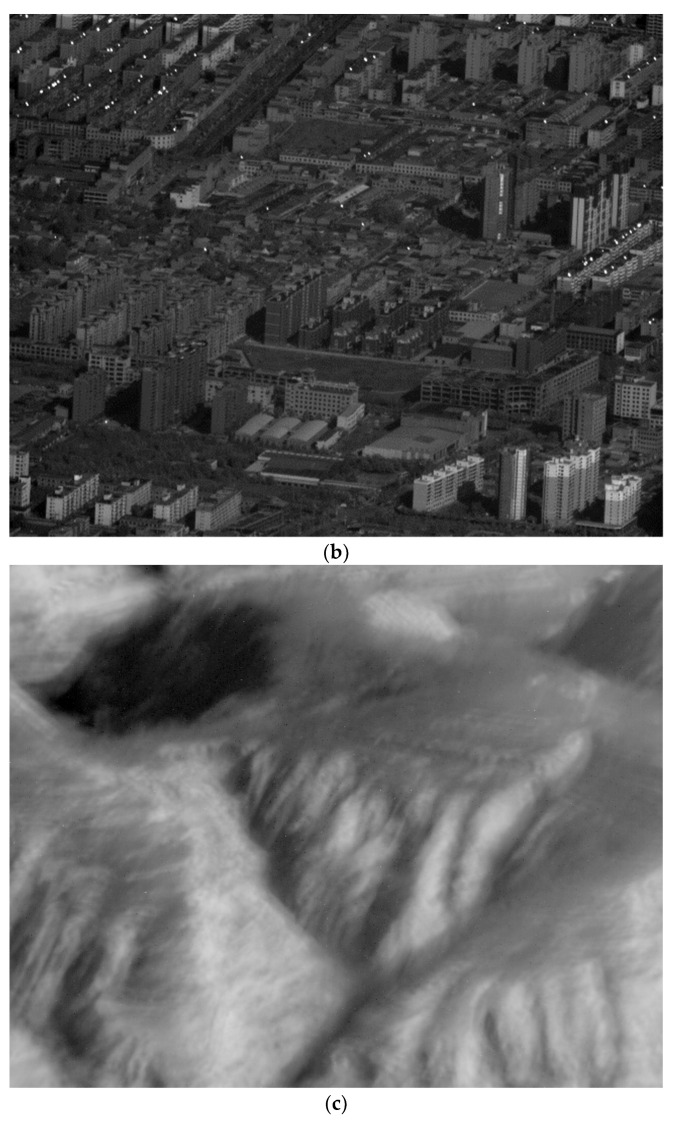

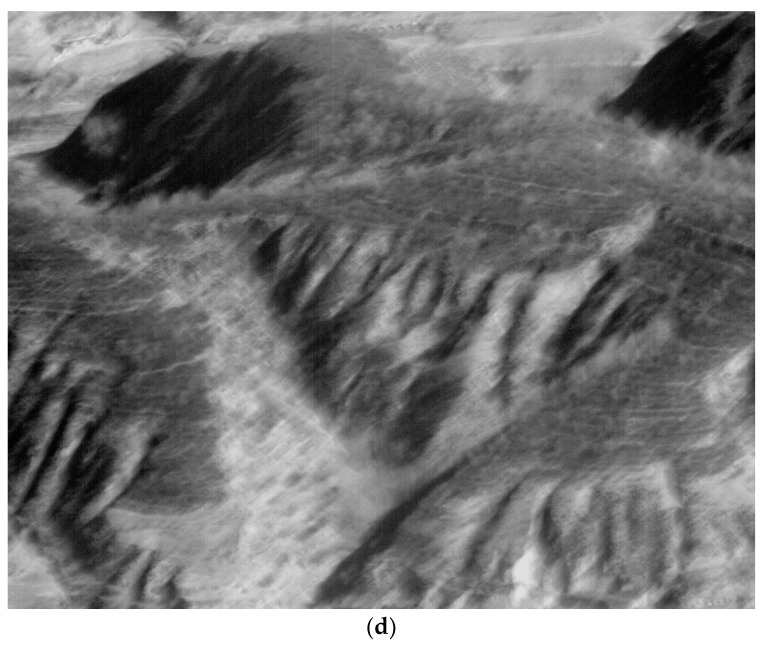
Images obtained from flight tests; (**a**) Infrared image with right IMC; (**b**) Visible light image with right IMC; (**c**) Infrared image with wrong IMC; (**d**) Visible light image with wrong IMC.

The imaging system is installed at the belly of the Y12 aircraft during the flight test. The visible light and infrared images obtained are shown in Figure 19. Figure 19a,b show the images when the IMC rates of the FSM were correct, and the images are clear; Figure 19c,d show the images when the IMC rates of the FSM were modified to the incorrect values by multiplying ${\dot{\delta }}_{T}$ and ${\dot{\lambda }}_{T}$ by 0.5. Consequently, the images are blurred. It can be proven that the FSMs create an effective compensation function and the compensation algorithm.

## 6. Conclusions

In this paper, a dynamic scan and stare imaging system is proposed, and two two-axis FSMs are used to compensate the image motion caused by the aircraft and gimbal angular velocity and the aircraft liner velocity. Both the structure and the mirror of the two-axis FSM adopt aluminum alloys, which effectively eliminate the reduction in mirror surface profile accuracy caused by the linear expansion coefficient mismatch. The RMS of the visible light mirror is 15.8 nm and that of the infrared mirror is 41.1 nm. The flexible support structure based on cross-axis flexible hinges is proposed, which has a large angle range of ±1.1°. The closed-loop bandwidth of the FSM is greater than 200 Hz. The IMC algorithm based on the coordinate transformation method is given. Finally, the effectiveness of the FSM and IMC algorithm is proven by the dynamic imaging test in the laboratory and flight test.

The dynamic scan and stare imaging system, the design and implementation scheme of the FSM, and the IMC algorithm proposed in this paper are universal and especially suitable for photoelectric equipment used for high-altitude and long-distance reconnaissance and surveillance.

## Figures and Tables

**Table 1:** Comparison of advantages and disadvantages of different imaging methods of aerial reconnaissance cameras.

Imaging Mode	Advantage	Disadvantage
**Staring imaging**	All the pixels of the detector are exposed at the same time; the imaging system itself will not introduce the image motion. The image motion can be reduced by shortening the exposure time [4].	In order to obtain wide-field-of-view images, a large focal plane array detector must be used. The imaging frame frequency of a large focal plane array detector is low, which makes it unfavorable for surveillance and tracking. A large focal plane array detector can easily cause image distortion [3,4,5,10].
**Linear array scanning imaging**	The imaging FOV can be expanded by scanning of the gimbal. TDI mode can be used to improve the signal-to-noise ratio (SNR) and realize IMC [3,8,9].	It cannot stare at specific areas for surveillance imaging [3,4,7].
**Step and stare imaging**	Based on area array detectors, the imaging FOV can be expanded by scan motion of the gimbal. The scanning stops at the moment of exposure [2,3,4,5,6]. Reconnaissance and surveillance can be realized at the same time.	Large rotational inertia of the gimbal leads to a long settling time of start/stop motion, which results in low step frame frequency, low scanning efficiency and limited expansion of the imaging FOV [2,3,4,5,6].
**Dynamic scan and stare imaging**	Due to the small focal plane array detector, the imaging frame frequency increases. Continuous scan motion of the gimbal enlarges the FOV. FSM is used to compensate the image motion caused by scan motion during the exposure [6,7,8,9]. Wide-area reconnaissance and target surveillance can be realized at the same time.	The structure of FSM, the compensation algorithm and the control method are complicated [6,7,8,9].

**Table 2:** Performing requirements of the FSM.

NO.	Characteristics	Performance
1	Optical aperture/mm	≥55×77.8 ^1^
2	Travel/°	±1.1
3	Angular resolution/μrad	5
4	Control bandwidth/Hz	≥200

^1^ The optical aperture is an ellipse and its minor axis length and major axis length are 55 mm and 77.8 mm, respectively.

**Table 3:** Results of mirror surface roughness/nm.

NO.	Roughness	NO.	Roughness
1	2.875	9	0.841
2	2.4	10	0.955
3	2.72	11	1.514
4	1.403	12	1.69
5	3.0	13	2.844
6	2.998	14	2.227
7	2.262	15	1.574
8	2.305	16	2.757

**Table 4:** Performances of linear voice coil motor.

NO.	Characteristics	Performance
1	Outline dimension	ϕ20×29 mm
2	Total stroke	4 mm
3	Clearance on side of coil	1 mm
4	Peak force	5.1 N
5	Weight of field assembly	14 g
6	Weight of coil assembly	20 g
7	Power @ Peak force	22 W

## Data Availability

The data and results used to support the research in this article can be obtained from the corresponding author.
